# Impact of partially defatted black soldier fly larvae meal on coccidia-infected chickens: effects on growth performance, intestinal health, and cecal short-chain fatty acid concentrations

**DOI:** 10.1186/s40104-025-01167-z

**Published:** 2025-02-26

**Authors:** Jing Yuan, Kolapo M. Ajuwon, Olayiwola Adeola

**Affiliations:** Department of Animal Sciences, Purdue University, West Lafayette, IN 47907 USA

**Keywords:** Black soldier fly larvae meal, Broiler chicken, Coccidia, Intestinal health

## Abstract

**Background:**

Black soldier fly larvae meal (BSFLM) stands out as a promising nutritional resource due to its rich bioactive substances and favorable protein profile. Nonetheless, its potential to mitigate coccidia infection in broilers remains uncertain. This study aimed to evaluate the impact of partially defatted BSFLM (pBSFLM) on growth performance, nutrient utilization, and intestinal health, focusing on morphology, immunology, and cecal fermentation in coccidia-infected broilers.

**Methods:**

Over the initial 13 d, 480 newly-hatched Cobb 500 male birds were allocated to three diets with increasing pBSFLM concentrations (0, 60, or 120 g/kg). At d 13 post hatching, chicks within each dietary group were further allotted to non-challenge or challenge subsets, generating six treatments in a 3 × 2 factorial arrangement. Challenged birds were orally administered oocysts of *E. maxima*, *E. acervulina*, and *E. tenella* (25,000:125,000:25,000).

**Results:**

During the infection phase (d 13 to 19), linear interactions between *Eimeria* and pBSFLM were observed in gain to feed ratio (G:F) (*P* < 0.05) and cecal interferon-γ (IFN-γ, *P* < 0.05), with a tendency in cecal acetate concentration (*P* = 0.06). A quadratic interaction was observed in crypt depth (CD, *P* < 0.05). Incremental pBSFLM inclusion negatively affected G:F, CD, IFN-γ, and acetate productions in the ceca under coccidia challenge. Conversely in non-challenged birds, the impact of pBSFLM varied from neutral (e.g. G:F) to potentially advantageous (e.g. acetate). Challenged birds exhibited decreased (*P* < 0.01) BW, BW gain, feed intake (FI), and the apparent ileal digestibility and total tract nutrient utilization of DM, gross energy, and nitrogen (N). *Eimeria* challenge reduced (*P* < 0.01) serum carotenoid concentrations, decreased the villus height to crypt depth ratio (VH:CD, *P* < 0.01), and increased concentrations of branched-chain fatty acids, specifically isobutyrate (*P* = 0.059) and isovalerate (*P* < 0.05) in the cecum. Dietary pBSFLM addition linearly reduced (*P* < 0.05) BW, FI, and N utilization. Tendencies (*P* < 0.06) were observed where pBSFLM linearly decreased VH:CD and reduced goblet cell density.

**Conclusions:**

Increasing pBSFLM supplementation, particularly at 12%, adversely affected growth, ileal morphology, cecal acetate production, and downregulated key cytokine expression in response to coccidia infection.

## Background

Optimal gastrointestinal health is crucial for animal production, directly impacting both health and performance [1]. The integrity of the gut barrier, composed of microbiota, mucus layer, and antimicrobial compounds, is fundamental to preventing the intrusion of harmful substances into the body [2]. However, *Eimeria* infection in poultry disrupts this barrier, causing significant damage to the intestinal mucosal cells [3, 4]. This breach leads to increased permeability, nutrient leakage, compromised digestion, and reduced protein absorption, manifesting in clinical and subclinical consequences of coccidiosis [5, 6]. Apart from its direct impact on epithelial cells, *Eimeria* invasion also disturbs the gut microbial communities, fostering the proliferation of pathogens like *Clostridium perfringens* [4]. Such disruptions trigger dysbiosis, an imbalance in the gut microbial community and its metabolism, thus undermining the gastrointestinal tract’s role in nutrient digestion and absorption and bird development [7]. Conventional coccidia treatment strategies, such as chemoprophylaxis and vaccines, have limitations due to the possibility of drug resistance and the potential selection for more virulent parasite strains under treatment pressure [4].

Insect meals, notably black soldier fly (BSF) larvae, are gaining prominence as alternative feed ingredients owing to their nutritional richness and eco-friendly rearing practices [8, 9]. Larvae of BSF, rich in protein and ether extract content, show promise in poultry feeding [8]. The dried larvae are replete with modulators of immunity and oxidative stress, alongside essential nutrients like amino acids, lipids, and vital minerals such as calcium, phosphorus, and iron. As the larvae progress in maturity, they develop into a distinctive reservoir of a chitosan-melanin complex that is covalently bound, comprising chitin, with an affinity for organic substances and heavy metals. In addition, their noteworthy melanin content delivers potent antioxidant effects, exhibiting antitumor, immunomodulatory, and antimicrobial properties [10]. The lipid fraction contains significant levels of lauric acid and its derivatives, known for their biological activity, converting into monolaurin with antiviral, antibacterial, and antiprotozoal attributes [11]. This varied array of components, encompassing hydrophobic substances, chitin, melanin, and lauric acid, makes the dried larva a potential treatment against viruses and bacteria. For this reason, there is a lot of interest in the use of black soldier fly as a component of animal feeds for its potential growth-promoting and health-related benefits. Additionally, non-defatted BSF larvae meal (BSFLM) in animal feed is prone to rapid rancidity, limiting its shelf life, while full defatting removes beneficial antimicrobial properties of medium-chain fatty acids (MCFAs) [12]. This study uses partially defatted BSFLM to retain key MCFAs, while reducing the risk of rancidity. Combined with components like chitin and melanin, partially defatted BSFLM (pBSFLM) offers both stability and health-promoting benefits as a feed additive. Hence, this study aimed to evaluate the effectiveness of including pBSFLM in the diets of broilers experimentally infected with coccidiosis.

## Materials and methods

### Broiler husbandry, experimental design, and dietary treatments

At hatch, 480 Cobb 500 male chicks (Siloam Springs, AR, USA) were individually weighed and tagged for identification purposes. Throughout the experiment, the chickens were accommodated in brooders featuring an electrically heated battery (model SB 4 T; Alternative Design Manufacturing & Supply, Siloam Springs, AR, USA). Employing body weight (BW) as a blocking factor, chicks were distributed to three dietary treatments using a randomized complete block design. In each dietary regimen, there were 16 replicate cages, all configured to house 10 chicks. On d 13 post hatching, individual chick weight was recorded, and subsequent groupings were conducted within the respective dietary treatments. Subsequently, a subset of 128 birds within each dietary treatment were selected and redistributed based on BW into two additional treatments, namely, challenge (CHA) and non-challenge (NCHA), with 8 birds per cage following a randomized complete block design. The chickens had ad libitum access to both feed and water the entire study, and daily mortality was recorded. The diets (Table 1) were fed in a mash form, comprising a corn-soybean meal base, and supplemented with partially defatted black soldier fly larvae meal (pBSFLM; Enterra Feed Co., Maple Ridge, BC, Canada) at 3 levels of 0, 60, or 120 g/kg. The nutritional composition of pBSFLM has been reported in a previous publication [12]. All experimental diets were formulated to meet or exceed the nutritional specifications recommended for Cobb 500 chicks [13]. Chromic oxide (5 g/kg) was added into all diets as an indigestible marker.

**Table 1:** Ingredients and calculated nutrient levels of the experimental diets, g/kg as-fed basis^a^

Item	Concentration of pBSFLM, g/kg		
	0	60	120
Ingredient, g/kg			
Corn	441.7	480.4	519.1
DDGS	91.0	91.0	91.0
pBSFLM	0.0	60.0	120.0
Soybean meal	339.2	266.6	194.0
Megalac	15.0	7.5	0.0
Soybean oil	50.6	32.8	15.0
Ground limestone	10.8	11.4	12.0
Monocalcium phosphate	13.8	12.4	11.0
Salt	4.0	4.0	4.0
L-Lysine HCl	2.0	2.1	2.2
DL-Methionine	2.9	2.9	3.0
L-Threonine	1.0	0.9	0.7
Vitamin-mineral premix^b^	3.0	3.0	3.0
Chromic oxide premix^c^	25.0	25.0	25.0
Total	1,000.0	1,000.0	1,000.0
Calculated nutrient, g/kg			
ME, kcal/kg	3,071.7	3,096.8	3,121.9
Crude protein	206.7	207.1	207.5
Ether extract	71.3	64.4	57.6
Calcium	8.8	8.7	8.7
Phosphorus	6.7	6.5	6.3
Non-phytate P	4.4	4.4	4.4
Amino acid (SID), %			
Arginine	1.1	1.1	1.0
Histidine	0.5	0.6	0.7
Isoleucine	0.7	0.7	0.7
Leucine	1.5	1.5	1.5
Lysine	1.0	1.0	1.0
Methionine	0.5	0.5	0.5
Methionine + Cysteine	0.8	0.8	0.8
Phenylalanine	0.8	0.8	0.8
Threonine	0.7	0.7	0.7
Tryptophan	0.2	0.2	0.2
Valine	0.8	0.8	0.9

^a^*Abbreviations*: *pBSFLM* Partially defatted black soldier fly larva meal, *DDGS* Distiller's dried grains with solubles, *SID* Standardized ileal digestibility

^b^Provided the following quantities per kg of complete diet: vitamin A, 10,968 IU; vitamin D_3_, 5,286 IU; vitamin E, 22.0 IU; menadione, 8.76 mg; riboflavin, 11.0 mg; D-pantothenic acid, 22.0 mg; niacin, 88.2 mg; choline chloride, 1,542 mg; vitamin B_12_, 0.03 mg; biotin, 0.11 mg; thiamine mononitrate, 4.40 mg; folic acid, 1.98 mg; pyridoxine hydrochloride, 6.60 mg; I, 2.22 mg; Mn, 132 mg; Cu, 8.88 mg; Fe, 88.2 mg; Zn, 88.2 mg; Se, 0.60 mg

^c^5 g Chromic dioxide plus 20 g corn

### Coccidia challenge and sampling procedures

On d 8, 12, and 19 post hatching, individual bird BW and cage feed consumption were measured to calculate BW gain, feed intake (FI), and gain to feed ratio (G:F). Birds were orally administered either a 1 mL solution containing oocysts of three selected *Eimeria species* (*E. maxima*:*E. tenella*:*E. acervulina* = 25,000:25,000:125,000) or a 1 mL phosphate buffer saline (PBS, VWR International, Radnor, PA, USA) on d 13 post hatching. To maintain sample integrity and prevent potential contamination with *Eimeria*, a strict protocol was observed during all collections, ensuring a sequential transition from the NCHA group to the CHA group and subsequent departure from the facility. Samples of excreta were collected using paper-lined pans positioned beneath individual cages over three consecutive days from d 17. Subsequently, these samples were preserved at −20 °C for later analysis to determine the apparent total tract utilization (ATTU) of nutrients. On d 19, chicks were humanely sacrificed by CO_2_ asphyxiation. Two birds with median body weight from each cage were selected for blood, mid-ileum, ceca, and cecal content sampling, while the remaining six out of eight chicks in each cage were used to obtain ileal digesta. Detailed procedures on collection and subsequent analysis of ileal digesta are in a previous study [14].

### Plasma collection and analysis

Blood samples were collected via cardiac puncture from a single bird in each cage following euthanasia by CO_2_ inhalation and were then deposited into EDTA tubes on ice. Subsequently, these tubes were centrifuged at 1,250 × *g* in 4 °C for 20 min. To facilitate further analysis, plasma from birds housed in a single cage were pooled, aliquoted and stored at −80 °C. All subsequent carotenoid analysis procedures were carried out under subdued yellow light conditions. Concentrations of carotenoid were measured using spectrophotometry, following the methodology previously detailed by Allen [15]. Additionally, alpha-1-acid glycoprotein (AGP) were quantified using a sandwich enzyme-linked immune absorbent assay (ELISA) kit, following the manufacturer guidelines (Life Diagnostics, Inc., West Chester, PA, USA).

### Intestinal morphology

The methodology outlined in the work by Alagbe et al. [16] served as the basis for the preparation of samples intended for intestinal morphometry analysis. In brief, approximately 10-cm intestinal segments were excised from mid-ileum, flushed in ice-cold 10% PBS, and fixed in 10% buffered formalin (VWR International, Radnor, PA, USA) for at least 30 d. Following this, three tissue sections, each measuring 4 mm in thickness, were procured from every sample. The Purdue Histology and Phenotyping Laboratory (Purdue University, West Lafayette, IN, USA) then carried out further processing and staining using Alcian blue and periodic acid-Schiff (AB-PAS). Measurements of villus height (VH) and crypt depth (CD) and counts of goblet cells (GC) were conducted utilizing a microscope (National Optical and Scientific Instruments, Inc. in Schertz, TX, USA), and quantified with ImageJ macro (ImageJ opensource software version 1.8). Nine villi per bird were selected for measurement, focusing solely on intact villi without signs of damage. The villus height to crypt depth (VH:CD) ratio was determined, while GC density was calculated as the ratio of GC count and VH.

### Ceca mucosal immunity and SCFA and BCFA levels

Cecal contents from selected birds were snap-frozen in liquid nitrogen and placed in −80 °C for subsequent extraction and analysis of short-chain fatty acids (SCFAs) and branched-chain fatty acids (BCFAs) following a previous protocol [17]. Approximately 0.2 g of cecal contents were mixed with 1 mL of deionized water, thoroughly homogenized, and then centrifuged at 14,000 × *g* for 10 min. Consequently, 400 μL of supernatant was obtained, which was subsequently combined with 100 μL of an internal standard comprising 4-methylvaleric acid, 85% phosphoric acid, and copper sulfate pentahydrate. The processed mixture was temporarily stored at −80 °C prior to the determination of SCFAs and BCFAs content using gas chromatography (GC-FID 7890A, Agilent, Santa Clara, CA, USA). The settings included an injector temperature set at 230 °C and a gradual increase in the oven temperature from 100 °C to 200 °C. For constructing standard curves, a gradient dilution was employed for the external standards comprising acetic acid, propionic acid, butyric acid, iso-butyric acid, and isovaleric acid. The concentrations (mmol/g) of individual SCFA and BCFA was derived from standard curves, modified using a dilution factor of 1.25 according to the protocol, and subsequently normalized by the weight of the extracted frozen cecal samples.

Simultaneously, cecal segments were longitudinally sectioned, rinsed three times with PBS, and the mucosal scrapings collected were snap-frozen in liquid nitrogen and placed in −80 °C until subsequent analysis. Mucosal scrapings were processed using TissueLyser equipment (Qiagen, Valencia, CA, USA). The concentration of interferon (IFN)-γ and interleukin (IL)-1β were determined using commercial ELISA kits from Thermo Fisher (Cambridge, MA, USA) and MyBioSource (San Diego, CA, USA), respectively. The standards and samples were subsequently analyzed in duplicate following the manufacturer’s guidelines.

### Statistical analysis

Data analyses were conducted using the MIXED procedure in SAS (SAS Inst. Inc., Cary, NC, USA). For evaluating the pre-*Eimeria* infection growth performance and nutrient digestibility, the model included the 3 diets as a fixed effect, with the 16 blocks (initial BW of chicks) as a random effect. For the subsequent post-infection phase of the study, a 3 × 2 factorial arrangement was adopted, incorporating the three levels of pBSFLM inclusion (0, 60, or 120 g/kg; or 0, 6%, or 12%) and the 2 *Eimeria* challenge states (CHA or NCHA) as main effects. The fixed effects comprised the 6 treatments (different combinations of pBSFLM levels and challenge states), and the random effects were the 8 blocks (initial BW of chicks). To explore linear and quadratic effects of increasing pBSFLM levels or the interactions between pBSFLM addition and challenge states, orthogonal polynomial contrasts were applied. Outliers were identified and removed based on the criterion of values falling outside 1.5-fold the interquartile range. Statistical significance was set at *P* < 0.05, tendency at *P*-value less than 0.10, and cage as the experimental unit.

## Results

### Growth performance, ileal digestibility, total tract nutrient utilization

The growth performance and utilization of nutrients in the pre-coccidia challenge period when fed increasing concentrations of pBSFLM during post hatching d 1 to 12 is shown in Table 2. A linear increase (*P* < 0.05) was observed in both BW gain and G:F during d 1 to 8 as pBSFLM levels rose in the chicken diets. However, the effects of pBSFLM supply during d 8 to 12 on growth performance was not significant (*P* > 0.05). In all, addition of pBSFLM linearly increased (*P* < 0.05) G:F of broiler chicken from d 1 to 12.

**Table 2:** Growth performance and total tract nutrient utilization of broiler chickens fed diets supplemented with 0, 6%, or 12% pBSFLM from d 1 to 12 post hatching^a^

Item	Concentration of pBSFLM, %			SEM	*P*-value^2^	
	0	6	12		L	Q
BW, g						
d 1	52	52	52	1.4	-	-
d 8	205	213	216	3.8	< 0.001	0.404
d 12	409	417	413	7.6	0.684	0.391
d 1 to 8						
BW gain, g/bird	152.4	160.1	162.5	2.63	0.001	0.265
Feed intake, g/bird	171.9	170.9	171.0	3.09	0.794	0.829
G:F, g/kg	890	937	951	11.9	0.001	0.250
d 8 to 12						
BW gain, g/bird	204.3	204.2	196.2	5.80	0.215	0.478
Feed intake, g/bird	248.9	249.1	237.9	7.84	0.193	0.429
G:F, g/kg	820	821	830	12.1	0.565	0.810
d 1 to 12						
BW gain, g/bird	357.1	364.8	360.4	6.97	0.684	0.392
Feed intake, g/bird	420.0	418.9	407.6	8.46	0.200	0.532
G:F, g/kg	850	871	887	9.9	0.012	0.806
Total tract nutrient utilization, %						
DM, %	74.3	74.6	74.8	0.68	0.598	0.926
GE, %	78.0	78.3	78.3	0.63	0.687	0.746
N, %	72.6	71.0	70.8	1.05	0.216	0.567
Number of observations	16	16	16			

^a^*Abbreviations*: *pBSFLM* Partially defatted black soldier fly larva meal, *G:F* Gain to feed ratio

^2^L, linear effect of pBSFLM; Q, quadratic effect of pBSFLM

A summary of the main effects and interactions between pBSFLM and challenge states on growth performance and nutrient utilization from post hatching d 13 to 19 is provided in Table 3. Four observations from the 0 g/kg pBSFLM within the challenge group were detected as outliers and were not included in the analyses. The addition of pBSFLM caused a linear reduction (*P* < 0.05) in BW gain and FI, despite the challenge states. Challenge reduced (*P* < 0.01) BW on d 19 for broiler chickens, and (*P* < 0.01) BW gain and FI. The apparent ileal digestibility (AID) and ATTU of N were linearly reduced (*P* < 0.05) by pBSFLM supplementation regardless of the challenge states. *Eimeria* challenge led to a decrease (*P* < 0.01) in the AID and ATTU of DM, GE, and N. An interaction between pBSFLM and challenge was found (*P* < 0.05) in G:F.

**Table 3:** Growth performance, apparent ileal digestibility, and total tract nutrient utilization of broiler chickens fed diets supplemented with 0, 6%, or 12% pBSFLM with or without *Eimeria* challenge from d 13 to 19 post hatching^a^

Item	Concentration of pBSFLM, %			Challenge		SEM	*P*-value				
							pBSFLM^b^		Challenge	pBSFLM × Challenge	
	0	6	12	NCHA	CHA		L	Q		L	Q
BW, g											
d 12	426	435	432	431	431	12.3	-	-	-	-	-
d 19	747	713	692	786	648	28.2	0.068	0.790	< 0.001	0.231	0.394
BW gain, g/bird	319.2	272.1	260.4	355.4	212.5	20.92	0.010	0.315	< 0.001	0.125	0.157
Feed intake, g/bird	445.3	397.3	396.9	469.9	356.3	22.91	0.041	0.237	< 0.001	0.821	0.366
G:F, g/kg	680	662	637	755	564	20.0	0.043	0.813	< 0.001	0.010	0.209
Apparent ileal digestibility											
DM, %	68.4	66.6	66.7	77.2	57.3	1.88	0.254	0.441	< 0.001	0.352	0.941
GE, %	68.1	66.4	66.5	79.8	54.2	1.93	0.306	0.482	< 0.001	0.492	0.803
N, %	77.5	76.0	74.1	85.5	66.2	1.82	0.032	0.995	< 0.001	0.351	0.231
Total tract nutrient utilization											
DM, %	72.8	72.9	72.8	74.8	70.8	0.79	0.768	0.700	< 0.001	0.197	0.432
GE, %	76.5	76.8	76.3	78.6	74.5	0.72	0.985	0.371	< 0.001	0.113	0.364
N, %	70.1	69.2	67.1	72.0	65.6	1.27	0.028	0.490	< 0.001	0.903	0.251
Number of observations	12	16	16	24	20						

^a^*Abbreviations*: *pBSFLM* Partially defatted black soldier fly larva meal, *NCHA* Non-challenge, *CHA* Challenge; *G:F* Gain to feed ratio, *GE* Gross energy, *N* Nitrogen

^b^L, linear effect of pBSFLM or interaction between pBSFLM and challenge; Q, quadratic effect of pBSFLM or interaction between pBSFLM and challenge

### Intestinal morphology

Results of the effect of pBSFLM inclusion and *Eimeria* challenge on the intestinal morphology, GC count and density are presented in Table 4. A quadratic interaction effect was observed (*P* < 0.05) for ileal crypt depth in response to different levels of BSF inclusion. Notably, at 6% BSF inclusion, the NCHA group showed the highest crypt depth, whereas the CHA group had the lowest. In the CHA group, crypt depth peaked at 12% BSF inclusion. Dietary supplementation with pBSFLM resulted in tendencies to linearly decrease the VH:CD ratio and GC density, despite the challenge states. Challenge led to deeper (*P* < 0.01) CD and a decrease (*P* < 0.01) in VH:CD ratio in the ileum.

**Table 4:** Histomorphology of broiler chickens fed diets supplemented with 0, 6%, or 12% pBSFLM with or without *Eimeria* challenge, d 19 post hatching^a^

Item	Concentration of pBSFLM, %			Challenge		SEM	*P*-value				
							pBSFLM^b^		Challenge	pBSFLM × Challenge	
	0	6	12	NCHA	CHA		L	Q		L	Q
VH, μm	432.3	451.4	445.2	434.4	451.6	43.84	0.816	0.755	0.443	0.106	0.148
CD, μm	90.8	96.6	107.8	87.4	109.4	7.72	0.029	0.565	0.001	0.088	0.018
VH:CD ratio	5.02	4.87	4.45	5.21	4.35	0.354	0.058	0.780	0.006	0.759	0.378
GC count, cells/villi	150.4	150.6	126.7	136.9	148.3	20.64	0.242	0.784	0.330	0.382	0.932
GC density, cells/μm of VH	0.34	0.34	0.29	0.31	0.33	0.028	0.052	0.356	0.296	0.569	0.311
Number of observations	15	13	16	21	23						

^a^*Abbreviations*: *pBSFLM* partially defatted black soldier fly larva meal, *NCHA* non-challenge, *CHA* challenge, *VH* villi height, *CD* crypt depth, *GC* goblet cell

^b^L, linear effect of pBSFLM or interaction between pBSFLM and challenge; Q, quadratic effect of pBSFLM or interaction between pBSFLM and challenge

### Plasma biomarkers and intestinal cytokines

The plasma concentrations of carotenoids and AGP, as well as cecal cytokines IL-1β and IFN-γ in chickens on d 19 post hatching are outlined in Table 5. No significant main effect of pBSFLM was observed on the concentration of carotenoids, AGP, and IL-1β. Coccidia challenge led to a decrease (*P* < 0.01) in the concentrations of plasma carotenoids and cecal IL-1β. Interaction between pBSFLM and challenge was observed (*P* < 0.01) in IFN-γ.

**Table 5:** Concentrations of cytokines of broiler chickens fed diets supplemented with 0, 6%, or 12% pBSFLM with or without *Eimeria* challenge, d 19 post hatching^a^

Item	Concentration of pBSFLM, %			Challenge		SEM	*P*-value				
							pBSFLM^b^		Challenge	pBSFLM × Challenge	
	0	6	12	NCHA	CHA		L	Q		L	Q
Carotenoids, μg/mL	2.8	3.1	2.9	4.0	1.9	0.29	0.815	0.324	< 0.001	0.489	0.126
AGP, μg/mL	134.0	152.8	175.4	147.1	161.1	28.55	0.434	0.769	0.357	0.792	0.852
IL-1β, ng/mL	1.2	1.7	1.7	2.6	0.5	0.63	0.419	0.551	< 0.001	0.843	0.835
IFN-γ, ng/mL	2.88	2.91	3.09	2.90	3.02	0.095	0.016	0.142	0.405	0.007	0.967
Number of observations	14	15	12	20	21						

^a^*Abbreviations*: *pBSFLM* partially defatted black soldier fly larva meal, *NCHA* non-challenge, *CHA* challenge, *AGP* alpha-1-acid glycoprotein, *IFN* interferon, *IL* interleukin

^b^L, linear effect of pBSFLM or interaction between pBSFLM and challenge; Q, quadratic effect of pBSFLM or interaction between pBSFLM and challenge

### Intestinal SCFA and BCFA

The impacts of pBSFLM and challenge on the concentrations of SCFAs and BCFAs in the ceca are summarized in Table 6. There were no linear or quadratic effects of pBSFLM alone on cecal SCFAs and BCFAs. Coccidia challenge decreased the concentration of total SCFAs (*P* < 0.01), while causing the concentration of isovalerate to increase (*P* < 0.05), with a tendency to elevate (*P* = 0.059) isobutyrate level. There was a tendency (*P* = 0.060) of linear interaction between pBSFLM and challenge observed in acetate.

**Table 6:** Levels of measured cecal SCFAs and BCFAs in mmol/g cecal content weight of broiler chickens fed diets supplemented with 0, 6%, or 12% pBSFLM with or without *Eimeria* challenge, d 19 post hatching^a^

Item	Concentration of pBSFLM, %			Challenge		SEM	*P*-value				
							pBSFLM^b^		Challenge	pBSFLM × Challenge	
	0	6	12	NCHA	CHA		L	Q		L	Q
Acetate	47.0	44.7	47.4	58.1	34.7	5.41	0.734	0.707	< 0.001	0.060	0.977
Propionate	5.7	5.4	5.5	5.1	6.0	0.70	0.904	0.862	0.180	0.157	0.678
Isobutyrate	1.4	1.2	1.2	1.1	1.5	0.18	0.521	0.602	0.059	0.504	0.112
Butyrate	10.1	8.6	9.8	9.6	9.4	1.72	0.736	0.542	0.598	0.523	0.575
Isovalerate	1.6	1.3	1.4	1.1	1.8	0.25	0.481	0.429	0.002	0.836	0.249
Total	65.8	61.3	65.4	75.0	53.4	7.55	0.780	0.650	< 0.001	0.102	0.857
Number of observations	15	16	16	24	23						

^a^*Abbreviations*: *pBSFLM* partially defatted black soldier fly larva meal, *NCHA* non-challenge, *CHA* challenge, *SCFA* short-chain fatty acid, *BCFA* branched-chain fatty acid

^b^L, linear effect of pBSFLM or interaction between pBSFLM and challenge; Q, quadratic effect of pBSFLM or interaction between pBSFLM and challenge

## Discussion

In this study, we explored the impact of pBSFLM supplementation in the diets of broiler chickens experimentally infected with *Eimeria*. Birds were mildly infected in this study as evidenced by the lack of any mortalities post-challenge.

During the pre-challenge period, especially in the first week post hatching, supplementing pBSFLM positively impacted the growth performance of broilers in the current trial, demonstrating improvements of up to 6% in terms of enhanced BW and G:F. The increased BW and G:F due to pBSFLM addition partially aligned with findings from Bellezza Oddon et al. [18], who observed 10% improvement in growth performance with increased BW and daily FI during the starter phase (d 1–10) with 10% defatted BSFLM inclusion. The high protein content and favorable amino acid profile of BSFLM make for a valuable feedstuff as was widely reported in pigs, chickens, and other poultry [19]. However, it is worth noting that there is an optimal inclusion rate for promoting weight gain and animal health [20]. The positive effects of BSFLM supplementation on growth might be attributed to increasing palatability and natural inclination of chickens to consume insects, which is further supported by the feed-choice test conducted by Cullere et al. [21]. Despite this, the presence of chitin, which is indigestible by monogastric animals, in BSFLM could negatively potentially impact protein digestibility, thereby further reducing feed efficiency [22]. Although previous research noted changes in feed consumption, our study did not show significant impact on this parameter. Nevertheless, these differences in both the extent of this variation and the overall enhancement in growth performance can be attributed to these factors: nutritional composition of the BSFLM utilized, which is affected by the developmental period (pupa, larva, or adult) of BSF, the environment where the insects were raised, the defatting procedure [23], and also the developmental stage of the chickens studied.

Multiple research investigations have highlighted the primary impacts of coccidiosis in broiler chickens by disrupting gut homeostasis, leading to substantial malabsorption, diminished feed conversion and BW gain, and productivity decline [24]. Aligning with our current findings, the 3-mixture *Eimeria* spp*.* reduced BW and FI in chickens. With increasing inclusion level of pBSFLM, the BW and FI linearly decreased regardless of challenge state. However, a finding not in consonance with previous knowledge is that pBSFLM supplementation notably reduced the feed efficiency ratio in chicks facing coccidia challenge, but no distinction was found in the absence of challenge. To the best of our knowledge, previous research on BSF has primarily focused on its optimal inclusion ratio, as well as its impact on growth and physiological indicators such as blood parameters, intestinal morphology [25], gut microbiota [26], meat quality [27], etc. These studies have predominantly explored normal physiological conditions or, in other words, healthy states. However, the impact of BSFLM supplementation on animal growth and physiological markers under pathological conditions has not been widely reported. The starter period holds paramount importance in broiler production, characterized by rapid growth and development, thereby significantly shaping subsequent growth rates. This prompted an investigation into the effects of introducing BSFLM during the early stages and subsequent exposure to coccidia challenge during the growing phase.

*Eimeria* challenge resulted in decreased AID and ATTU of DM, N, and energy, aligning with prior research indicating that coccidia challenge reduced utilization of nutrient in broilers [16, 18]. The decrease in nutrient absorption may partly account for the observed reduction in growth performance in birds exposed to coccidiosis. Dietary pBSFLM provision led to a decrease in N digestibility in broilers irrespective of their state of challenge, consequently leading to an excessive influx of undigested nitrogen into the ceca. This influx may potentially impact microbial metabolism and fermentation within the ceca, along with overall health status of the ceca. Subsequently, this may be reflected in the expression of inflammatory cytokines and concentrations of cecal SCFAs. Existing literature indicated that the exoskeleton of partially defatted BSFLM contained a chitin amount ranging from 50 to 96 g/kg DM [28, 29]. Even though chickens produce chitinase in the oxyntico-peptic cells and hepatocytes, the breakdown of chitin appears to be suboptimal. The linearly decreased N utilization might be ascribed to the chitin content gradually introduced through BSFLM. The chitin present in exoskeleton has been reported to hinder nutrient absorption within the intestinal tract, consequently diminishing the absorption of both fat and protein in broilers [30]. Of particular note is the trend observed wherein the nitrogen utilization in the group fed pBSFLM declined further upon coccidia infection. It has been widely shown that coccidia infection significantly impacts the activity and expression of digestive enzymes [31], as well as excessive endogenous loss [32], leading to reduced N utilization. While the influence of coccidia infection on the activity and expression of chitinase remains unclear, chitin itself, as an indigestible fiber, under the condition of coccidia infection, potentially exacerbated endogenous nitrogen losses, consequently further reducing nitrogen utilization. Thus, this might partially elucidate the underlying reason for the reduced nitrogen utilization trend observed in the pBSFLM-fed group under coccidia infection.

The evaluation of avian performance has evolved to include an assessment of gut health dynamics. A healthy, optimally functioning intestine is widely acknowledged as a fundamental indicator of animal well-being and a primary determinant of overall growth in poultry [1]. Specifically, the morphometric analysis of specific gut segments and mucosal elements, such as crypts and villi, in conjunction with blood chemistry, has emerged as a reliable and accessible approach for concurrently characterizing both the intestinal health and the broader health status of broilers [33]. In this study, *Eimeria* challenge was observed to induce villus atrophy (data not shown), thickened the lamina propria, evident in deeper crypts, and decreased VH:CD ratio. These alterations, known to impede digestion and nutrient absorption, were linked to subsequent reductions in growth performance [34]. More importantly, increasing inclusion of pBSFLM in the diet, especially 12% pBSFLM, worsened the adverse impacts of coccidia infection, leading to a notable increase in crypt depth. Enlarged crypts could be associated with heightened cell turnover, with implications of a greater energy demand for gut maintenance. This leads to the utilization of nutrients for the functionality of the digestive tract rather than for growth [35]. Prior studies have predominantly emphasized the impact of 15% or higher inclusion of BSFLM on intestinal morphology, noting deeper crypts and reduced VH:CD ratio in the jejunum and ileum of broilers [25] and laying hens [36] under healthy conditions, with recommendations for lower BSFLM inclusion levels. The current study revealed that the addition of 12% pBSFLM did not influence crypt depth in the intestines under coccidia-free conditions. However, in the presence of coccidia infection, the adverse effects of high concentrations of pBSFLM suggest that when utilizing pBSFLM as a feed additive, considerations should be given to its use in diseased conditions. Furthermore, dietary pBSFLM inclusion tended to reduce VH:CD ratio and goblet cell density regardless of the challenge state. Goblet cells are specialized cells secreting glycoproteins that aid in purging foreign pathogens and preventing detrimental substances from directly interacting with the intestinal epithelium [37]. In the current study, incorporation of pBSFLM resulted in a tendency to compromise ileal morphology, resulting in a decrease in goblet cell density, with potential interference with glycoprotein secretion. Consequently, these events may disrupt the intestinal mucosal immune response and induce changes in the intestinal microenvironment, thereby exacerbating the adverse effects of coccidia infection.

Several blood biomarkers were employed to gauge the severity of *Eimeria* infection in the current experiment. Plasma carotenoids, an indirect, yet swift indicator, offer significant potential for detecting avian intestinal damage caused by coccidiosis, even at low oocyst inoculation levels with minimal performance impact [38]. In our study, a significant decline in plasma carotenoids post-coccidiosis further affirmed the successful establishment of the coccidia model and indicated intestinal damage, aligning with the morphological findings. A hepatically synthesized acute phase protein, α1-acid glycoprotein, that is responsive to pro-inflammatory cytokines upon detecting *Eimeria* invasion within the innate immune system, serves as a marker for systemic inflammation in poultry. Chen et al. [39] reported a 3.8-fold change in AGP levels in Ross 308 broilers following coccidiosis vaccine challenge. However, the current observation of a lack of response in plasma AGP to coccidia challenge in broilers aligns with the findings of Rochell et al. [38]. The heterogeneous response of AGP across various coccidiosis challenge experiments may be closely associated with factors such as diet composition, chicken breed, challenge dose, *Eimeria* species, detection timings, and the assay kit’s sensitivity. Moreover, Goossens et al. [40] emphasized that elevated circulating acute phase protein (APP) levels may lack specificity for gut barrier failure, as non-gastrointestinal inflammatory diseases could also raise serum APPs. Consequently, we further extended our investigation by evaluating the local immune response through an assessment of cytokine expression within the ceca.

In the sampling process, observable and notable hemorrhaging was observed in nearly all ceca of the coccidia-infected group fed with 12% pBSFLM. Consequently, a detailed analysis of the immune response within the ceca was conducted. *Eimeria* parasites, upon invading epithelial cells, prompt diverse and complex immune reactions, and incite local inflammatory responses in broilers. Cytokines, essential immune regulatory peptides, play a pivotal role in mediating protective immunity against these parasites and serve as crucial biomarkers for assessing intestinal health. The expression of IFN-γ serves as a prevalent indicator of cellular immunity against avian coccidiosis, orchestrated by CD4 ^+^ and CD8 ^+^ effector lymphocytes [41]. Previous studies have demonstrated that *E. tenella* induced CD4 ^+^ T cell activation, initiated a macrophage response, and upregulated IFN-γ expression in the cecum, leading to bolstered protective immunity against *Eimeria* challenge [42]. In non-challenge group, there was a linear increase in cecal IFN-γ concentration as the pBSFLM inclusion increased. However, under coccidiosis, as pBSFLM addition rose, the fluctuation in IFN-γ was less significant. The response of cecal IFN-γ to pBSFLM addition varied depending on challenge or non-challenge state. The lower IFN-γ expression under coccidiosis due to the gradual pBSFLM inclusion indicated an impaired immune response against coccidiosis. The prevailing immune response in avian coccidiosis was thought to be the T helper (Th)1 response mediated by IFN-γ [43]. Significant anticoccidial effects by hindering parasite invasion and survival within host cells is exerted by IFN-γ. It promotes local inflammation, stimulates nitric oxide production, activates cell-mediated cytotoxicity, and facilitates the release of cytoplasmic granules containing perforin and proteases [44]. The decreased concentrations of IFN-γ subsequently reduced the defense against coccidiosis in the current experiment. de Souza Vilela et al. [45] observed the decreased CD3 ^+^ T lymphocytes and cytotoxic T lymphocytes CD3 ^+^ CD8 ^+^ in jejunum with increasing full-fat BSFLM supplementation up to 20% BSFLM in broilers under healthy physiological conditions, which led to the hypothesis the potential immunomodulatory effects of BSFLM might stem from its fatty acid profile, along with the expression of antimicrobial peptides and chitin, establishing an efficient antimicrobial barrier. This could potentially diminish the need for heightened intraepithelial cytotoxic T lymphocyte. A previous study found that 9% full-fatted BSFLM increased sIgA and decreased IFN-γ level in ileal mucosa in healthy laying hens [46]. Two other studies highlighted the beneficial effects of 3% or 10% BSFLM supplementation on stimulating nonspecific immune responses by improving the T lymphocytes proliferation in spleens against *Salmonella gallinarum* and coronavirus in broilers, respectively [47, 48]. The existing research on the impact of BSFLM on the immune system of broilers is notably limited, particularly on the immune status during disease states, with a scarcity of studies focusing on the local intestinal immune response. Discrepancies among various studies’ findings could be attributed to the composition of BSFLM (e.g., full-fat or partially defatted), sampling sites, disease models, broiler age, to name a few. Subsequent studies are imperative to investigate the precise mechanisms underlying the protective effects of BSFLM in broiler chicks. Furthermore, a comprehensive understanding of the primary component(s) within BSFLM responsible for its immune-enhancing effects is essential, given the complex array of compounds present in BSFLM.

The cytokine IL-1β plays a crucial role in initiating inflammation. Hong et al. [49] observed an increase in IL-1β expression during the initial hours following infection with *E. tenella* and *E. maxima*. Contrarily, our study identified a notable decrease in IL-1β expression in the ceca, this might be associated with the expression of anti-inflammatory factors, like IL-10 [50], which inhibit the synthesis of proinflammatory cytokines. Another possibility could be the timing of the sampling, potentially occurring during the later stages of the inflammatory response. The decreased concentrations of IL-1β in this case might indicate a prior intense immune response in the ceca following coccidia infection.

Furthermore, we tested the impacts of pBSFLM administration on SCFA concentration in the ceca. SCFAs, such as butyrate, acetate, valerate, and propionate, are mainly generated from fermenting indigestible carbohydrates. Conversely, BCFAs such as isobutyrate and isovalerate arise from protein or amino acid fermentation [51]. The profiles of SCFAs mirror intestinal fermentation patterns and microbial activity, pivotal for gut health and enterocyte functions [52]. In the current study, coccidia challenge significantly diminished the levels of total SCFAs while elevating the presence of isovalerate. Furthermore, there were discernible increase in the concentrations of isobutyrate as well. This observation is consistent with prior research outcomes [53, 54], which can be an indicator of a shift in the ratio of protein and carbohydrate substrates available for fermentation in the hindgut or a potential alteration in hindgut environment. The higher BCFAs in the ceca of chickens challenged with *Eimeria* could be adduced to two reasons. Firstly, the diminished N utilization and compromised intestinal morphology might lead to reduced protein absorption in the small intestine, resulting in protein accumulation in the hindgut. Secondly, substantial quantities of epithelial cell debris generated due to intestinal damage induced by coccidiosis could serve as fermentable protein substrates, ultimately reaching the ceca. Interestingly, in the absence of challenge, there is a progressive increase in acetate levels with the gradual increase in dietary pBSFLM supplementation. However, with coccidia challenge, the addition of pBSFLM conversely led to a declining trend in acetate levels. In line with our findings, Borrelli et al. [55] showed a similar outcome where BSFLM supplementation enriched bacterial chitinase levels in the ceca. This enrichment facilitated the deacetylation of chitin into acetate, resulting in elevated acetate levels observed in healthy laying hens. Under the condition of coccidia challenge, there was reversal of the anticipated increase in acetate concentration. This reversal might be attributed to a substantial influx of undigested proteins into the hindgut, promoting the proliferation of protein-degrading microbial communities. This shift could potentially supplant chitin fermentation or, alternatively, the abundance of fermentation byproducts from proteins might overwhelm the efficacy of chitin fermentation.

## Conclusions

This study further unraveled the impacts of pBSFLM supplementation on broilers under coccidia challenge and the data demonstrated that while pBSFLM supplementation at certain levels could have positive impacts on the growth and well-being of healthy broilers, when broilers are subjected to coccidia infection, previously considered safe or beneficial levels may also elicit adverse effects on growth, intestinal morphology, immunity, and hindgut fermentation. Consequently, in production settings, these potential adverse impacts should not be overlooked or disregarded. Furthermore, the presence of harmful pathogens in BSF larvae rearing substrates may lead to potential pathogen transmission. Therefore, it is crucial to implement rigorous processing and careful diet formulation, considering the physiological or health status of the animals, before incorporation of insect-based ingredients into poultry diets to ensure the health of the birds is not compromised.

## Data Availability

All data from this study are available from the corresponding author upon reasonable request.

## Abbreviations

AGP: Alpha-1-acid glycoprotein
AID: Apparent ileal digestibility
ATTU: Apparent total tract utilization
BCFA: Branched-chain fatty acid
BSF: Black soldier fly
BW: Body weight
CD: Crypt depth
CHA: Challenge
FI: Feed intake
G:F: Gain to feed ratio
GC: Goblet cells
IFN: Interferon
IL: Interleukin
MCFA: Medium-chain fatty acid
NCHA: Non-challenge
pBSFLM: Partially defatted black soldier fly larvae meal
SCFA: Short-chain fatty acid
VH: Villus height
VH:CD: Villus height to crypt depth ratio

## References

[1] Kogut MH, Arsenault RJ. Editorial: Gut health: The new paradigm in food animal production. Front Vet Sci. 2016;3:71. 10.3389/fvets.2016.00071. 27630994 10.3389/fvets.2016.00071 PMC5005397

[2] Broom LJ. Gut barrier function: Effects of (antibiotic) growth promoters on key barrier components and associations with growth performance. Poult Sci. 2018;97:1572–8. 10.3382/ps/pey021. 29462405 10.3382/ps/pey021

[3] Nabian S, Arabkhazaeli F, Seifouri P, Farahani A. Morphometric analysis of the intestine in experimental coccidiosis in broilers treated with anticoccidial drugs. Iran J Parasitol. 2018;13:493–9. https://www.ncbi.nlm.nih.gov/pmc/articles/PMC6243159/. 30483343 PMC6243159

[4] Yang C, Kennes YM, Lepp D, Yin X, Wang Q, Yu H, et al. Effects of encapsulated cinnamaldehyde and citral on the performance and cecal microbiota of broilers vaccinated or not vaccinated against coccidiosis. Poult Sci. 2020;99:936–48. 10.1016/j.psj.2019.10.036. 32029170 10.1016/j.psj.2019.10.036 PMC7587813

[5] Quiroz-Castañeda RE, Dantán-González E. Control of avian coccidiosis: Future and present natural alternatives. BioMed Res Int. 2015;2015:e430610. 10.1155/2015/430610. 10.1155/2015/430610 PMC4346696 25785269

[6] Leung H, Yitbarek A, Snyder R, Patterson R, Barta JR, Karrow N, et al. Responses of broiler chickens to *Eimeria* challenge when fed a nucleotide-rich yeast extract. Poult Sci. 2019;98:1622–33. 10.3382/ps/pey533. 30481335 10.3382/ps/pey533 PMC6414034

[7] Ducatelle R, Eeckhaut V, Haesebrouck F, Immerseel FV. A review on prebiotics and probiotics for the control of dysbiosis: present status and future perspectives. Animal. 2015;9:43–8. 10.1017/S1751731114002584. 10.1017/S1751731114002584 25336177

[8] Makkar HPS, Tran G, Heuzé V, Ankers P. State-of-the-art on use of insects as animal feed. Anim Feed Sci Technol. 2014;197:1–33. 10.1016/j.anifeedsci.2014.07.008.

[9] Meneguz M, Schiavone A, Gai F, Dama A, Lussiana C, Renna M, et al. Effect of rearing substrate on growth performance, waste reduction efficiency and chemical composition of black soldier fly (*Hermetia illucens*) larvae. J Sci Food Agric. 2018;98:5776–84. 10.1002/jsfa.9127. 29752718 10.1002/jsfa.9127

[10] Bastrakov AI, Dontsov AE, Ushakova NA. The black soldier fly *Hermetia illucens* under artificial breeding conditions is a renewable source of the melanin-chitosan complex. Izvestia Ufimskogo Nauchnogo Tsentra RAN. 2016;4:77–9.

[11] Omores RA. Extraction, fractionation, nanoparticles formulation, and antimicrobial activity of lipids from black soldier fly larvae. PhD thesis. Cape Town: University of the Western Cape; 2021.

[12] Osunbami OT, Adeola O. Regression method-derived digestible and metabolizable energy concentrations of partially defatted black soldier fly larvae meal for broiler chickens and pigs. Livest Sci. 2022;264:105042. 10.1016/j.livsci.2022.105042.

[13] Vantress C. Cobb500 broiler performance and nutrition supplement. 2018; https://www.cobbafrica.com/wp-content/uploads/Cobb-500-Broiler-Management-Supplement.pdf.

[14] Yuan J, Johnson TA, Ajuwon KM, Adeola O. *Eimeria* infection-related intestinal dynamics and microbiome, growth performance, and nutrient utilization in broiler chickens fed diets supplemented with multienzyme. Can J Anim Sci. 2023;103:81–91. 10.1139/cjas-2022-0046.

[15] Allen PC. Effect of *Eimeria acervulina* infection on chick (*Gallus domesticus*) high density lipoprotein composition. Comp Biochem Physiol B. 1987;87:313–9. 10.1016/0305-0491(87)90145-3. 3621900 10.1016/0305-0491(87)90145-3

[16] Alagbe EO, Schulze H, Adeola O. Growth performance, nutrient digestibility, intestinal morphology, cecal mucosal cytokines and serum antioxidant responses of broiler chickens to dietary enzymatically treated yeast and coccidia challenge. J Anim Sci Biotechnol. 2023;14:57. 10.1186/s40104-023-00846-z. 37038240 10.1186/s40104-023-00846-z PMC10084602

[17] Tuncil YE, Thakkar RD, Marcia ADR, Hamaker BR, Lindemann SR. Divergent short-chain fatty acid production and succession of colonic microbiota arise in fermentation of variously-sized wheat bran fractions. Sci Rep. 2018;8:16655. 10.1038/s41598-018-34912-8. 30413754 10.1038/s41598-018-34912-8 PMC6226458

[18] Bellezza Oddon S, Biasato I, Imarisio A, Pipan M, Dekleva D, Colombino E, et al. Black soldier fly and yellow mealworm live larvae for broiler chickens: Effects on bird performance and health status. J Anim Physiol Anim Nutr. 2021;105:10–8. 10.1111/jpn.13567. 10.1111/jpn.13567 PMC8519120 34402110

[19] Abd El-Hack ME, Shafi ME, Alghamdi WY, Abdelnour SA, Shehata AM, Noreldin AE, et al. Black soldier fly (*Hermetia illucens*) meal as a promising feed ingredient for poultry: A comprehensive review. Agriculture. 2020;10:339. 10.3390/agriculture10080339.

[20] Sverguzova SV, Shaikhiev IH, Sapronova ZA, Fomina EV, Makridina YL. Use of fly larvae *Hermetia illucens* in poultry feeding: a review paper. J Water Land Dev. 2021;95–103. 10.24425/jwld.2021.137101.

[21] Cullere M, Tasoniero G, Giaccone V, Miotti-Scapin R, Claeys E, Smet SD, et al. Black soldier fly as dietary protein source for broiler quails: apparent digestibility, excreta microbial load, feed choice, performance, carcass and meat traits. Animal. 2016;10:1923–30. 10.1017/S1751731116001270. 10.1017/S1751731116001270 27339654

[22] Sánchez-Muros M-J, Barroso FG, Manzano-Agugliaro F. Insect meal as renewable source of food for animal feeding: a review. J Clean Prod. 2014;65:16–27. 10.1016/j.jclepro.2013.11.068.

[23] Barragan-Fonseca KB, Dicke M, van Loon JJA. Nutritional value of the black soldier fly (*Hermetia illucens* L.) and its suitability as animal feed – a review. J Insects Food Feed. 2017;3:105–20.

[24] Freitas LFVB de, Sakomura NK, Reis M de P, Mariani AB, Lambert W, Andretta I, et al. Coccidiosis infection and growth performance of broilers in experimental trials: insights from a meta-analysis including modulating factors. Poult Sci. 2023;102:103021. 10.1016/j.psj.2023.103021. 37666145 10.1016/j.psj.2023.103021 PMC10491763

[25] Dabbou S, Gai F, Biasato I, Capucchio MT, Biasibetti E, Dezzutto D, et al. Black soldier fly defatted meal as a dietary protein source for broiler chickens: Effects on growth performance, blood traits, gut morphology and histological features. J Anim Sci Biotechnol. 2018;9:49. 10.1186/s40104-018-0266-9. 30002825 10.1186/s40104-018-0266-9 PMC6036674

[26] Biasato I, Ferrocino I, Dabbou S, Evangelista R, Gai F, Gasco L, et al. Black soldier fly and gut health in broiler chickens: insights into the relationship between cecal microbiota and intestinal mucin composition. J Anim Sci Biotechnol. 2020;11:11. 10.1186/s40104-019-0413-y. 32025297 10.1186/s40104-019-0413-y PMC6996183

[27] Dabbou S, Lauwaerts A, Ferrocino I, Biasato I, Sirri F, Zampiga M, et al. Modified black soldier fly larva fat in broiler diet: Effects on performance, carcass traits, blood parameters, histomorphological features and gut microbiota. Animals. 2021;11:1837. 10.3390/ani11061837. 34205603 10.3390/ani11061837 PMC8233813

[28] Kroeckel S, Harjes A_GE, Roth I, Katz H, Wuertz S, Susenbeth A, et al. When a turbot catches a fly: Evaluation of a pre-pupae meal of the black soldier fly (*Hermetia illucens*) as fish meal substitute — Growth performance and chitin degradation in juvenile turbot (*Psetta maxima*). Aquaculture. 2012;364–365:345–52. 10.1016/j.aquaculture.2012.08.041.

[29] Schiavone A, De Marco M, Martínez S, Dabbou S, Renna M, Madrid J, et al. Nutritional value of a partially defatted and a highly defatted black soldier fly larvae (*Hermetia illucens* L.) meal for broiler chickens: apparent nutrient digestibility, apparent metabolizable energy and apparent ileal amino acid digestibility. J Anim Sci Biotechnol. 2017;8:51. 10.1186/s40104-017-0181-5. 28603614 10.1186/s40104-017-0181-5 PMC5465574

[30] Khempaka S, Chitsatchapong C, Molee W. Effect of chitin and protein constituents in shrimp head meal on growth performance, nutrient digestibility, intestinal microbial populations, volatile fatty acids, and ammonia production in broilers. J Appl Poult Res. 2011;20:1–11. 10.3382/japr.2010-00162.

[31] Su S, Miska KB, Fetterer RH, Jenkins MC, Wong EA. Expression of digestive enzymes and nutrient transporters in *Eimeria*-challenged broilers. Exp Parasitol. 2015;150:13–21. 10.1016/j.exppara.2015.01.003. 25617757 10.1016/j.exppara.2015.01.003

[32] Teng P-Y, Yadav S, Shi H, Kim WK. Evaluating endogenous loss and standard ileal digestibility of amino acids in response to the graded severity levels of *E. maxima* infection. Poult Sci. 2021;100:101426. 10.1016/j.psj.2021.101426. 34547620 10.1016/j.psj.2021.101426 PMC8463777

[33] Biasato I, Gasco L, De Marco M, Renna M, Rotolo L, Dabbou S, et al. Yellow mealworm larvae (*Tenebrio molitor*) inclusion in diets for male broiler chickens: effects on growth performance, gut morphology, and histological findings. Poult Sci. 2018;97:540–8. 10.3382/ps/pex308. 29121342 10.3382/ps/pex308

[34] Tsukahara T, Inoue R, Nakayama K, Inatomi T. Inclusion of *Bacillus amyloliquefaciens* strain TOA5001 in the diet of broilers suppresses the symptoms of coccidiosis by modulating intestinal microbiota. Anim Sci J. 2018;89:679–87. 10.1111/asj.12980. 29282825 10.1111/asj.12980

[35] Williams J, Mallet S, Leconte M, Lessire M, Gabriel I. The effects of fructo-oligosaccharides or whole wheat on the performance and digestive tract of broiler chickens. Br Poult Sci. 2008;49:329–39. 10.1080/00071660802123351. 18568758 10.1080/00071660802123351

[36] Cutrignelli MI, Messina M, Tulli F, Randazzo B, Olivotto I, Gasco L, et al. Evaluation of an insect meal of the black soldier fly (*Hermetia illucens*) as soybean substitute: Intestinal morphometry, enzymatic and microbial activity in laying hens. Res Vet Sci. 2018;117:209–15. 10.1016/j.rvsc.2017.12.020. 29304440 10.1016/j.rvsc.2017.12.020

[37] Kim JJ, Khan WI. Goblet cells and mucins: Role in innate defense in enteric infections. Pathogens. 2013;2:55–70. 10.3390/pathogens2010055. 25436881 10.3390/pathogens2010055 PMC4235714

[38] Rochell SJ, Parsons CM, Dilger RN. Effects of *Eimeria acervulina* infection severity on growth performance, apparent ileal amino acid digestibility, and plasma concentrations of amino acids, carotenoids, and α1-acid glycoprotein in broilers. Poult Sci. 2016;95:1573–81. 10.3382/ps/pew035. 26933234 10.3382/ps/pew035

[39] Chen J, Tellez G, Richards JD, Escobar J. Identification of potential biomarkers for gut barrier failure in broiler chickens. Front Vet Sci. 2015;2:14. 10.3389/fvets.2015.00014. 10.3389/fvets.2015.00014 PMC4672187 26664943

[40] Goossens E, Debyser G, Callens C, De Gussem M, Dedeurwaerder A, Devreese B, et al. Elevated faecal ovotransferrin concentrations are indicative for intestinal barrier failure in broiler chickens. Vet Res. 2018;49:51. 10.1186/s13567-018-0548-4. 29925427 10.1186/s13567-018-0548-4 PMC6011339

[41] Yun CH, Lillehoj HS, Choi KD. *Eimeria tenella* infection induces local gamma interferon production and intestinal lymphocyte subpopulation changes. Infect Immun. 2000;68:1282–8. 10.1128/iai.68.3.1282-1288.2000. 10678939 10.1128/iai.68.3.1282-1288.2000 PMC97280

[42] Zhang DF, Xu H, Sun BB, Li JQ, Zhou QJ, Zhang HL, et al. Adjuvant effect of ginsenoside-based nanoparticles (ginsomes) on the recombinant vaccine against *Eimeria tenella* in chickens. Parasitol Res. 2012;110:2445–53. 10.1007/s00436-011-2784-7. 22215190 10.1007/s00436-011-2784-7

[43] Shah MAA, Song X, Xu L, Yan R, Song H, Ruirui Z, et al. The DNA-induced protective immunity with chicken interferon gamma against poultry coccidiosis. Parasitol Res. 2010;107:747–50. 10.1007/s00436-010-1940-9. 20574837 10.1007/s00436-010-1940-9

[44] Gomez-Osorio LM, Dehaeck B, Cuello C, Chaparro-Gutierrez JJ, Lopez-Osorio S, Gomez-Osorio LM, et al. From understanding the immune response against coccidiosis to the use of coccidia vaccines. Poult Farming-New Perspect Appl. IntechOpen; 2023. 10.5772/intechopen.110611.

[45] de Souza Vilela J, Andronicos NM, Kolakshyapati M, Hilliar M, Sibanda TZ, Andrew NR, et al. Black soldier fly larvae in broiler diets improve broiler performance and modulate the immune system. Anim Nutr. 2021;7:695–706. 10.1016/j.aninu.2020.08.014. 34466674 10.1016/j.aninu.2020.08.014 PMC8379420

[46] Chu X, Li M, Wang G, Wang K, Shang R, Wang Z, et al. Evaluation of the low inclusion of full-fatted *Hermetia illucens* larvae meal for layer chickens: Growth performance, nutrient digestibility, and gut health. Front Vet Sci. 2020;7:585843. 10.3389/fvets.2020.585843. 10.3389/fvets.2020.585843 PMC7728616 33330711

[47] Lee J, Kim Y-M, Park Y-K, Yang Y-C, Jung B-G, Lee B-J. Black soldier fly (*Hermetia illucens*) larvae enhances immune activities and increases survivability of broiler chicks against experimental infection of *Salmonella* Gallinarum. J Vet Med Sci. 2018;80:736–40 10.1292/jvms.17-0236. 29657236 10.1292/jvms.17-0236 PMC5989015

[48] Zhang Y, Yang C-Y, Li C, Xu Z, Peng P, Xue C, et al. Black soldier fly (*Hermetia illucens* L.) larval diet improves CD8^+^ lymphocytes proliferation to eliminate chicken coronavirus at an early infection stage. Vet Microbiol. 2021;260:109151. 10.1016/j.vetmic.2021.109151. 34237662 10.1016/j.vetmic.2021.109151

[49] Hong YH, Lillehoj HS, Lee SH, Dalloul RA, Lillehoj EP. Analysis of chicken cytokine and chemokine gene expression following *Eimeria acervulina* and *Eimeria tenella* infections. Vet Immunol Immunopathol. 2006;114:209–23. 10.1016/j.vetimm.2006.07.007. 10.1016/j.vetimm.2006.07.007 16996141

[50] Groux H, Powrie F. Regulatory T cells and inflammatory bowel disease. Immunol Today. 1999;20:442–5. 10.1016/S0167-5699(99)01510-8. 10500290 10.1016/s0167-5699(99)01510-8

[51] Tan J, McKenzie C, Potamitis M, Thorburn AN, Mackay CR, Macia L. Chapter three - The role of short-chain fatty acids in health and disease. In: Alt FW, editor. Adv Immunol. Academic Press; 2014. p. 91–119. 10.1016/B978-0-12-800100-4.00003-9. 10.1016/B978-0-12-800100-4.00003-9 24388214

[52] Polansky O, Sekelova Z, Faldynova M, Sebkova A, Sisak F, Rychlik I. Important metabolic pathways and biological processes expressed by chicken cecal microbiota. Appl Environ Microbiol. 2016;82:1569–76. 10.1128/AEM.03473-15. 10.1128/AEM.03473-15 PMC4771310 26712550

[53] Lin Y, Teng P-Y, Olukosi OA. The effects of xylo-oligosaccharides on regulating growth performance, nutrient utilization, gene expression of tight junctions, nutrient transporters, and cecal short chain fatty acids profile in *Eimeria*-challenged broiler chickens. Poult Sci. 2022;101:102125. 10.1016/j.psj.2022.102125. 36088820 10.1016/j.psj.2022.102125 PMC9468463

[54] Lin Y, Lourenco JM, Olukosi OA. The effects of protease, xylanase, and xylo-oligosaccharides on growth performance, nutrient utilization, short-chain fatty acids, and microbiota in *Eimeria*-challenged broiler chickens fed low-protein diet. Poult Sci. 2023;102:102789. 10.1016/j.psj.2023.102789. 37354614 10.1016/j.psj.2023.102789 PMC10404748

[55] Borrelli L, Coretti L, Dipineto L, Bovera F, Menna F, Chiariotti L, et al. Insect-based diet, a promising nutritional source, modulates gut microbiota composition and SCFAs production in laying hens. Sci Rep. 2017;7:16269. 10.1038/s41598-017-16560-6. 29176587 10.1038/s41598-017-16560-6 PMC5701250

